# New Molecular Geometries with Pentagonal-Pyramidal Structure

**DOI:** 10.1021/acsomega.5c07780

**Published:** 2025-12-03

**Authors:** Ricardo R. Oliveira, Wania Wolff, Amir L. Perlin

**Affiliations:** † Chemistry Institute, 28125 Federal University of Rio de Janeiro, Rio de Janeiro, RJ 21941-909, Brazil; ‡ Physics Institute, 28125 Federal University of Rio de Janeiro, Rio de Janeiro, RJ 21941-909, Brazil

## Abstract

In this work, we propose the design of molecular ions that present the same structure as the benzene dication, which has a pentagonal–pyramidal geometry. The chosen systems were obtained by replacing atoms or functional groups in C_6_H_6_^2+^ to form other species with eight valence electrons. They are C_5_H_6_Si^2+^, C_5_H_5_N^2+^, C_5_H_5_P^2+^, C_5_H_6_B^+^, and C_5_H_6_Al^+^. Applying a global minimum (GM) search combined with DFT computations, we were able to obtain GM candidates for all of the cases. Except for the pyridine dication, all GM candidates for the ions exhibit a pentagonal-pyramidal geometry, indicating that eight valence electrons stabilize systems with penta- or hexacoordinated main group elements, completely analogous to the benzene case. Based on the analysis of our results, we encourage the experimental realization and laboratory detection of the phosphorine dication in a similar way to the original work on the detection of the low-energy isomers of benzene dications.

## Graphical abstract

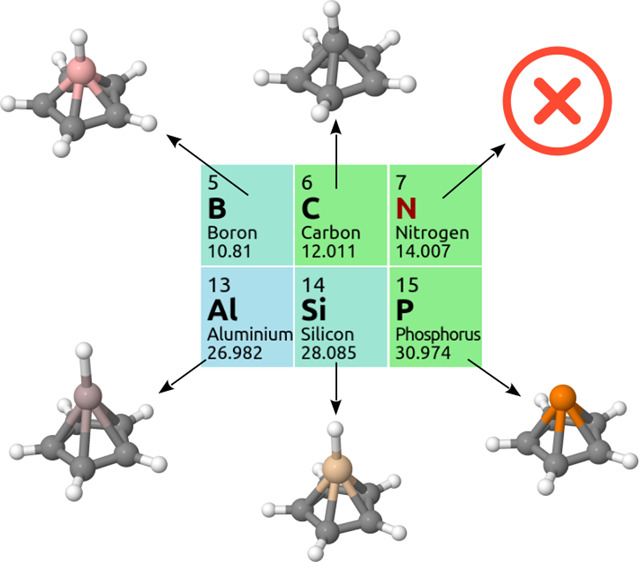

## 1 Introduction

The study of molecules and ions is an intense research field through the formation of exotic or complex molecular systems. It is possible to form and identify different isomers through molecular spectroscopy techniques.^1–5^ Even for larger molecules such as coronene (C_24_H_12_)^6^ and C_40_H_10_ nano bowl,^7^ which are molecular building blocks for fullerenes and nanotubes, the gas phase synthesis and characterization were successfully performed.^6,7^

In superacid media, it is possible to synthesize persistent hydrocarbon polycations.^8,9^ Thus, carbocations are important reaction intermediates for several transformations. These species can be formed by carbon and hydrogen rearrangement.^9,10^ Organic dications and pyramidal cations have been investigated for decades by applying experimental techniques in solution and in the gas phase assisted by computational methods.^8,11^ However, experimental realization and structure confirmation is challenging. A case of successes was the benzene dication^4,5,12^ but several organic pyramidal cations and dications remain elusive to date.^8,11^

Such systems are very important for astrochemistry and molecular spectroscopy, in particular, in obtaining high-resolution spectra to assist laboratory and interstellar medium (ISM) detections. For instance, different fullerene cations were detected, such as C_60_^+^, C_60_H^+^, and C_70_H^+^,^13–15^ and other protonated fullerenes were proposed.^14–17^ Polycyclic aromatic hydrocarbons (PAHs), which have similar infrared (IR) emission profiles of fullerenes, can also be synthesized and detected.^18–20^

Since the structure determination of benzene dication in the gas^4,5,21–24^ and condensed phase phase,^12^ which is pentagonal-pyramidal geometry, several efforts were made in order to reveal the structure of other ions with unusual chemical pattern. The nature of chemical bonding in this species was proposed by Fantuzzi et al.^25^ featuring a dative bond from C_5_H_5_^–^ anion to CH^3+^ cation, indicating a nonplanar hexacoordinate carbon atom. Molecules with planar hexacoordinated carbon atoms were recently proposed^26,27^ highlighting the interest in exotic molecular structures.

Unexpectedly, the most stable structure of the toluene dication exhibits a six-membered carbon ring. A hydrogen migration from the CH_3_ group to the meta position occur stabilizing this species.^28,29^ For the case of small PAHs, biphenyl^30^ and naphthalene,^31^ none of them presents a hexacoordinated carbon in the global minimum structures, indicating that the benzene dication has a unique molecular geometry. Even for phenanthrene there is no low-lying energy isomer with a hexacoordinate carbon atom.^32^ Other intriguing results were reported for the dications structures of monocyclic aromatic molecules. Aniline^33^ and chlorobenzene^34^ present a six-membered ring and a cyclopropenylium-allylidene structures, respectively.

The search of low-energy isomers and atomic clusters is an important topic in the field of theoretical chemistry, resulting in several different approaches and codes. Global optimizations based on genetic algorithms were developed in the last years, such as the famous Birmingham Cluster Genetic Algorithm (BCGA)^35^ and adapted codes.^36,37^ Also, other groups developed their own codes in order to have full control of the global optimization processes. Some examples are Gradient Embedded Genetic Algorithm (GEGA), Parallel Differential Evolution algorithm for Clusters Optimization (PDECO), CLUSTER, AUTOMATON, Global Optimizer of Clusters, Interfaces, and Adsorbates (GOCIA) and GAMaterial.^38–42^ Another coding strategy is based on the swarm intelligence optimization algorithm. An example is the suite of programs implemented in the ABCluster package, which allows different types of optimization coupled with several codes that allow calculations at different levels of theory.^43–46^

We can raise a few questions from the above discussion: Is it possible to form other pentagonal-pyramidal structures based on main-group elements? If yes, what combination of charge states and atoms will allow such a geometry to be stable enough to be formed and detected? The aim of the present work is to try to answer to this puzzle by applying theoretical chemistry methods, that is, combining the genetic algorithm implemented in the flexible and efficient AUTOMATON code^41,42^ and density functional theory (DFT) to find new structures in which main group elements present hypercoordination. The proposal was based on a simple hypothesis, hypercoordination can be obtained for systems similar to the benzene dication containing the same number of valence electrons.

It is important to mention that one motivation for the present work is the chemical challenge in order to stabilize pentagonal-pyramidal structures based on main-group elements. Since 1991, the pentagonal-pyramidal benzene dication was proposed as a global minimum by Krogh–Jespersen.^21^ Experimental confirmation in the gas phase at low temperature was provided by Jašík and co-workers based on infrared spectroscopy in an ion trap and in the solid state by Malischewski and Seppelt.^4,5,12^ In the present work we want to suggest new pentagonal-pyramidal systems for experimental realization in gas and condensated phases.

## 2 Computational Details

The search for the global minimum (GM) candidates in different electronic charge states forming eight valence electron systems was performed for C_5_H_6_B^+^, C_5_H_6_Al^+^, C_5_H_6_Si^2+^, C_5_H_5_N^2+^, and C_5_H_5_P^2+^. We included C_6_H_6_^2+^ for benchmark reasons. Due to the amount of geometries for each stoichiometry, the AUTOMATON software was used.^41,47^ This code associates a probabilistic cellular automaton method with a genetic algorithm (GA) and DFT through Gaussian 09 software.^48^ The Gaussian 09 software is used for geometry optimizations with no restrictions.

In short, a probabilistic cellular automaton method is used to construct the initial population. The atoms are positioned in regions that take into account their covalent radii. No distance smaller than the covalent radius of hydrogen will be considered. This strategy is quite effective for building reasonable molecular structures in the initial population. During the global optimization cycles, mating and mutation genetic operations were applied. Global convergence is obtained after nine cycles where there are no changes in the GM candidate. However, after each cycle, several molecular structures are similar. Due to this reason, a similarity check algorithm based on the comparison of distances is used to avoid repeated structures.

The parameters were chosen based on benchmark computations present in our previous work,^23^ with an initial population of 7N individuals, where N represents the number of atoms of the system. The default initial population of 5N individuals is not great enough in order to obtain the pentagonal–pyramidal geometry.^23^ Also, the PBE0 functional^49,50^ was used based on the AUTOMATON original work and results obtained by our group.^23,41,51^

Furthermore, the proposed basis set in the AUTOMATON original article, Stuttgart-Dresden effective core potential (ECP) with its corresponding double-ζ basis set,^52^ is not suitable to find the pentagonal-pyramidal geometry GM.^23^ Based on our previous results,^23,51^ we applied the and 6-31G­(d,p) basis set instead.

To obtain a better description of the low-lying isomers, the first ten low-lying energy isomers were selected for new optimization calculations. We reoptimized the isomers, employing the same functional but with the triple-ζ correlation consistent basis set family,^53,54^ cc-pVTZ.^55^ These calculations also included the zero-point energy (ZPE) and the harmonic frequencies, indicating if the structure is a minimum in the potential energy surface (PES). Thereafter, a single point computation was performed at the CCSD­(T) level of theory, using the same basis sets. All reported energies were ZPE-corrected CCSD­(T) ones. For each isomer, we employed the notation of the form **X.n.m**. The “**X**” represents the atomic symbol, “**n**” represents the energetic (final) order and “**m**” the charge state (+1 or +2).

## 3 Results and Discussion

### 3.1 C_6_H_6_^2+^ and C_5_H_6_Si^2+^

The computations for C_6_H_6_^2+^ low-energy isomers were performed previously^23^ but final energies were obtained only at the DFT level of theory. In this work, our results combined DFT and CCSD­(T) levels of theory and confirmed the energy order for the first isomers, which are present in Figure 1. The GM candidate (**C.1.2**) is the symmetrical pyramid with a cyclopentadienyl (C_5_H_5_^–^) as a base interacting with a CH^3+^ through an acceptor/donor bond (pentagonal-pyramidal structure).^25^ The top of the pyramid is centered and equidistant from the other carbon atoms. Since these results were obtained for benchmark reasons, we will not discuss further details.

**Figure 1:**
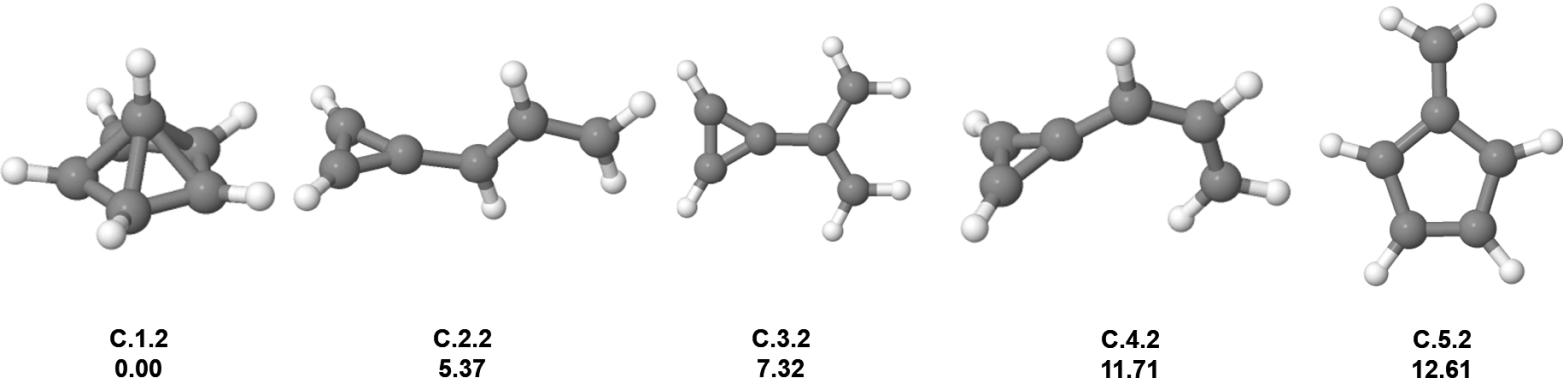
Low-lying isomers of C_6_H_6_^2+^. The first information above each structure is the isomer label: atomic symbol, energy order (first number), and charge state (second number). The second information is the relative energy in kcal mol^–1^. Carbon atoms are gray, and hydrogen atoms are white.

Changing a carbon atom for a silicon one, silabenzene dication is obtained, C_5_H_6_Si^2+^. The low-energy isomers are listed in Figure 2. The GM candidate (**Si.1.2**) is the fully symmetric pyramidal geometry with the cap being composed of SiH^3+^, similar to the benzene case, and the apical fragment is also equidistant from the carbon atoms. The second low-energy isomer (**Si.2.2**) is a new geometry which was not obtained for the benzene dication, where the cap hydrogen migrates to the ring, moving the carbon to a position below the base plan. Despite the reduction in geometry symmetry, the energy increase was only 5.91 kcal mol^–1^. This result indicates a change in the distribution of low-energy isomers when compared with the case of benzene. This structural change can be mapped by spectroscopic techniques of the silabenzene dication, in a similar fashion to the experiments carried out by Jašík et al.^4^ Experimental realization is a challenge because silabenzene (neutral) is an elusive metastable reaction intermediate.^56^ The relative energy gap of 11.87 kcal mol^–1^, between **Si.2.2** and **Si.3.2**, shows the preference of the pyramidal arrangement comparing to other geometries, including linear conjugated systems as **Si.5.2**. In **Si.3.2**, a cyclopentenium-like carbocation structure (c-C_5_H_7_^+^) where the carbon charge is delocalized within three carbon atoms, was obtained. The **Si.4.2** has 6.14 kcal mol^–1^ above **Si.3.2**. Note that for the case of benzene dication, the five-membered ring species (**C.5.2**) has the fulvene-like structure, different from **Si.3.2**. Concerning the **Si.5.2** system, a linear carbon chain is present, indicating a double bond conjugation.

**Figure 2:**
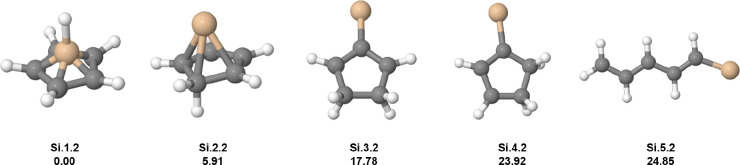
Low-lying isomers of C_5_H_6_Si^2+^. The first information above each structure is the isomer label: atomic symbol, energy order (first number), and charge state (second number). The second information is the relative energy in kcal mol^–1^. Carbon atoms are gray, hydrogen atoms are white and silicon atoms are yellow.

### 3.2 C_5_H_5_N^2+^ and C_5_H_5_P^2+^

Exchanging a CH group for a nitrogen atom in benzene dication, we have C_5_H_5_N^2+^, with 8 valence electrons. All of the isomers are present in Figure 3. The GM candidate (**N.1.2**) does not exhibit the pentagonal-pyramidal structure but a cyclopropenylium ring bonded to a linear carbon chain (similar to **C.2.2**) and a CNH group (protonated nitrile). This result is consistent with the hypothesis that steric effects are responsible for the instability of a pentacoordinated nitrogen favoring decomposition pathways.^57,58^ Formation of protonated nitriles was reported in cations of organic systems containing carbon, hydrogen, and nitrogen (acetonitrile and pyrrole cations).^51,59^ The **N.3.2** is 10.47 kcal mol^–1^ above the GM candidate, which has a similar structure but with a hydrogen migration to the nitrogen atom. **N.2.2** (which is 8.93 kcal mol^–1^ above the GM candidate) also has a cyclopropenylium group but is an isonitrile (NCH group). The fourth isomer (**N.4.2**) exhibits a six-membered ring where the nitrogen atom is protonated. This structure can be formed from the pyridine geometry through a hydrogen migration from the para position. It is worth to mention that aniline dication also has a six-membered ring (c-C_4_H_4_N–H) bonded to a CH_2_ group.^33^ The last isomer (**N.5.2**) is 19.21 kcal mol^–1^ above the GM candidate and is a cyclopropenylium connected to a N–C–CH_3_ linear chain.

**Figure 3:**
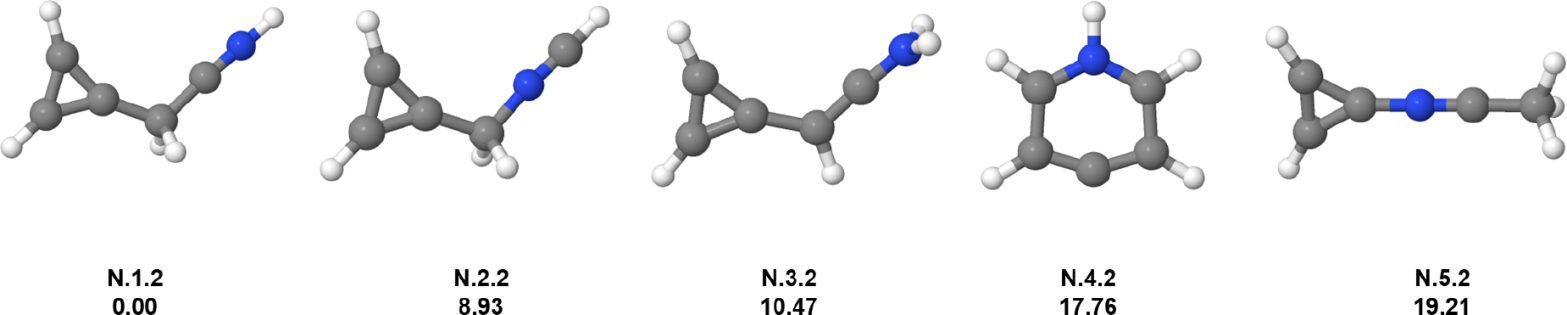
Low-lying isomers of C_5_H_5_N^2+^. The first information above each structure is the isomer label: atomic symbol, energy order (first number), charge state (second number). The second information is the relative energy in kcal mol^–1^. Carbon atoms are gray, hydrogen atoms are white, and nitrogen atoms are blue.

The phosphorine dication (C_5_H_5_P^2+^) is an analogue to pyridine dication, and the GM candidate has the pyramidal structure, similar to benzene and silabenzene GM candidates (see Figure 4). It is worth noting that the second isomer (**P.2.2**) is 37.11 kcal mol^–1^ above the GM candidate. This large difference in energy indicates that the GM candidate of phosphorine dication can be formed and trapped in an ion trap without much influence from other isomers. Consequently, this dication is a good candidate for experimental realization. Differently to the **N.1.2** structure, the **P.2.2** is consisted by H–C_2_P ring bonded to a carbon chain, i.e., the heteroatom forms the three-membered ring. **P.3.2** and **P.4.2** are very close to **P.2.2**. The last isomer, **P.5.2**, also has a pentagonal pyramidal geometry, but with the phosphorus atom inserted into the five-atom ring.

**Figure 4:**
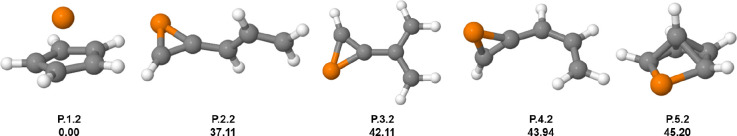
Low-lying isomers of C_5_H_5_P^2+^. The first information above each structure is the isomer label: atomic symbol, energy order (first number), charge state (second number). The second information is the relative energy in kcal mol^–1^. Carbon atoms are gray, hydrogen atoms are white, and phosphorus atoms are orange.

### 3.3 C_5_H_6_B^+^ and C_5_H_6_Al^+^

The borabenzene cation (C_5_H_6_B^+^), with 8 valence electrons, can be obtained by the exchange of a carbon atom for a B^–^ in the benzene dication. The lowest-energy (**B.1.1**) cation also has the pentagonal-pyramidal geometry, similar to dications of benzene, silabenzene, and phosphorine (see Figure 5). The second isomer (**B.2.1**) above 31.19 kcal mol^–1^ has a six-membered ring with two hydrogen atoms in the para position. **B.3.1** has a similar structure to the second hydrogen atom but in the ortho position. The fourth isomer (**B.4.1**) is composed of five carbon atoms ring bonded to a BH group, and it is 41.10 kcal mol^–1^ above the GM candidate. The last isomer (**B.5.1**) also has a five-membered ring, but with the boron atom composing the ring. Remarkably, all low-energy isomers have a cyclic structure containing five- or six-membered atoms. The large energy difference between **B.1.1** and **B.2.1** indicates that the borabenzene GM candidate can be efficiently formed in an ion trap, encouraging experimental detection. However, borabenzene has not been synthesized to this date; only the boratabenzene anion was obtained, indicating an experimental challenge.^60^

**Figure 5:**
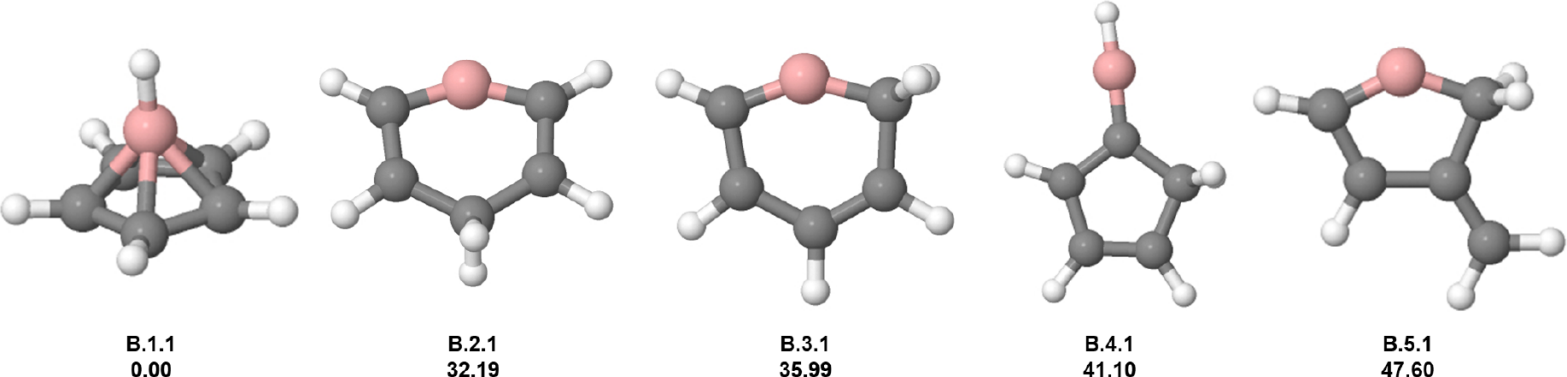
Low-lying isomers of C_5_H_6_B^+^. The first information above each structure is the isomer label: atomic symbol, energy order (first number), and charge state (second number). The second information is the relative energy in kcal mol^–1^. Carbon atoms are gray, hydrogen atoms are white, and boron atoms are pink.

Concerning C_5_H_6_Al^+^, which is analogue to C_5_H_6_B^+^, the GM candidate (**Al.1.1**) also has a pentagonal pyramidal structure. **Al.2.1** has a pyramid conformation with the hydrogen from the peak migrating to the ring, presenting a 18.53 kcal mol^–1^ above the GM candidate (see Figure 6). This structure is similar to that of **Si.2.2**. **Al.3.1** is 23.17 kcal mol^–1^ above the second isomer, very similar to the **B.4.1**. **Al.3.1** and **Al.4.1** have a similar structure, a five-membered carbon ring attached to a protonated aluminum atom. The last isomer (**Al.5.1**) has a completely different geometry arrangement with a carbon atom chain bonded to the Al atom. Compared to the boron case, the aluminabenzene anion was successfully synthesized,^61^ but the neutral counterpart remains elusive.

**Figure 6:**
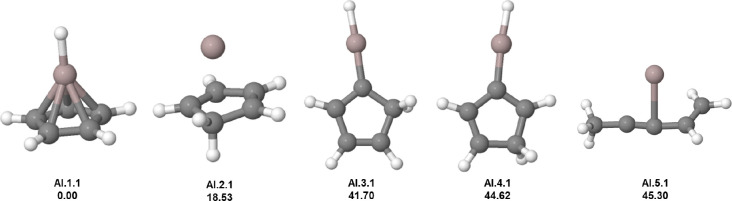
Low-lying isomers of C_5_H_6_Al^+^. The first information above each structure is the isomer label: atomic symbol, energy order (first number), and charge state (second number). The second information is the relative energy in kcal mol^–1^. Carbon atoms are gray, hydrogen atoms are white, and aluminum atoms are purple.

## 4 Kinetic Analysis

Considering the formation of pentagonal-pyramidal structures from six-membered rings, we performed a transition state search. Table 1 presents activation and reaction energies from six-membered rings to pentagonal-pyramidal isomerization reactions. The only isomerization that is not favorable is the pyridine dication reaction. The C_5_H_5_P^2+^ has the lowest activation energy, indicating that GM formation of this dication is promising. The C_5_H_6_B^+^ and C_5_H_6_Al^+^ have activation energies of 20.3 and 23.0 kcal mol^–1^, respectively, demonstrating that both species may have similar formation rates.

**Table 1:** Activation and Reaction Energies from Six-Membered Ring to Pentagonal-Pyramidal Isomerization

	activation energy (kcal mol^–1^)	reaction energy (kcal mol^–1^)
C_5_H_6_Si^2+^	28.6	–41.3
C_5_H_5_N^2+^	31.7	21.0
C_5_H_5_P^2+^	16.7	–63.4
C_5_H_6_B^+^	20.3	–52.9
C_5_H_6_Al^+^	23.9	–65.8

## 5 General Features of IR Spectra

### 5.1 C_5_H_6_Si^2+^

The simulated infrared spectra of C_5_H_6_Si^2+^ are present in Figure S1. In the spectrum of **Si.1.2**, only one strong band is present in the C–H stretching region (above 3000 cm^–1^), around 3200 cm^–1^. A band associated with the Si–H stretching mode is around 2250 cm^–1^. At lower wavenumbers, a few bands related to C–C stretching and C–C–C/C–C–H bending are also present in the spectra. The second isomer (**Si.2.2**) has three C–H stretching bands above 3000 cm^–1^, allowing differentiation of it from the lowest energy isomer. Isomers **Si.3.2** and **Si.4.2** have similar spectra due to the similar structure, with several bands below 2000 cm^–1^. The last isomer (**Si.5.2**) has a very different spectrum profile with intense bands in the C–C stretching and C–C–C/C–C–H bending regions, below 2000 cm^–1^.

### 5.2 C_5_H_5_N^2+^

All simulated infrared spectra of C_5_H_5_N^2+^ are present in Figure S2. In the spectrum of **N.1.2**, only one strong band is present due to the N–H stretching (around 3580 cm^–1^). The spectra of **N.2.2** and **N.3.2** have similar band profiles with several intense bands above 3000 cm^–1^ related to the C–H/N–H stretching modes. Also, intense bands are present between 2000 and 2500 cm^–1^ related to the C–N stretching mode. It is worth noting that the C–N stretching mode is much less intense in the GM candidate spectrum, turning this band a spectral fingerprint. For the case of **N.4.2**, only two intense bands appear above 3000 cm^–1^. For the fifth isomer (**N.5.2**), the most intense band is present around 2390 cm^–1^ due to the C–N stretching mode.

### 5.3 C_5_H_5_P^2+^

All simulated infrared spectra of C_5_H_5_P^2+^ are present in Figure S3. In the spectrum of the lowest-energy isomer (**P.1.2**), only one intense band is present due to the C–H stretching (around 3200 cm^–1^), very similar to the spectrum of **P.5.2**. The next three isomers, **P.2.2**, **P.3.2,** and **P.4.2**, have similar spectra with intense bands around 1500 cm^–1^ related to the C–C stretching and C–C–H bending modes. In conclusion, an intense band around 3200 cm^–1^ in the GM candidate (**P.1.2**) can be a fingerprint of the pentagonal-pyramidal structure, and all isomers with a three-membered ring present fingerprint bands around 1500 cm^–1^, which could be useful to detect these species.

### 5.4 C_5_H_6_B^+^

All simulated infrared spectra of C_5_H_6_B^+^ are present in Figure S4. In the spectrum of **B.1.1**, two intense bands are present due to the C–H out-of-plane distortion (around 1040 cm^–1^) and C–H stretching (around 3270 cm^–1^). The next two isomers, **B.2.1** and **B.3.1**, have similar and complex spectra profiles with intense bands around 1500 cm^–1^ related to the C–C stretching and C–C–H bending modes and above 3000 cm^–1^ due to C–H stretching modes. For the case of **B.4.1**, the most intense band is also around 1500 cm^–1^, but in this case, the C–H stretching bands are very weak. The last isomer (**B.5.1**) has a spectrum similar to the **B.2.1** and **B.3.1** ones.

### 5.5 C_5_H_6_Al^+^

All simulated infrared spectra of C_5_H_6_Al^+^ are present in Figures S5. In the spectrum of the GM candidate (**Al.1.1**), the most intense band is present below 1000 cm^–1^ due to the C–H out-of-plane distortion (around 890 cm^–1^). The second isomer (**Al.2.1**) has three intense bands below 1500 cm^–1^ due to C–C–C bending and C–C stretching modes. Also, two more bands are present in the C–H stretching region (around and above 3000 cm^–1^). The third (**Al.3.1**) and fourth (**Al.4.1**) isomers have similar spectra with several bands around and below 1500 cm^–1^. Finally, the last isomer (**Al.5.1**) has the most intense band around 2280 cm^–1^ due to the C–C stretching mode, where one carbon atom is bonded to the aluminum one.

## 6 Conclusions

We proposed new pentagonal-pyramidal molecular structures applying a global minimum search through a genetic algorithm combined with DFT and coupled cluster computations.^23,41^ The most stable structure of cations and dications of molecular complexes similar to the C_6_H_6_^2+^ has the pentagonal-pyramidal geometry; the only exception was the pyridine dication. The new candidates for GM molecular structures maintain eight valence electrons. For the elements from group 15 (nitrogen and phosphorus), the isoelectronic substitution was made from the CH group to the N or P atom. Further, for group 13, the substitution was from C to B^–^ or Al^–^. Preserving the same number of valence electrons, the lowest possible substitution using main group elements has proven to be an efficient strategy for stabilizing pentagonal-pyramidal structures. Remarkably, one main-group atom presents a hypercoordination bond situation.

The GM candidate for silabenzene is similar to the benzene dication case. The second isomer in energy also has a pentagonal-pyramidal geometry, and it is only 5.91 kcal mol^–1^ above. In the case of pyridine dication, there are no hypercoordination situations among the low-energy structures. However, for the phosphorine dication, the GM candidate exhibits a phosphorus atom in a hypercoordination situation, and the second isomer is 37.11 kcal mol^–1^ above the GM candidate. The borabenzene cation also presents a hypercoodination situation (boron atom) and has a 32.19 kcal mol^–1^ energy gap to the second isomer. Further, it is very similar for the aluminum case, in which the energy gap to the second isomer is 18.53 kal mol^–1^. In general, low-energy isomers of group 13 dications exhibit a preference for cyclic molecular geometry differently when compared to groups 14 and 15.

Considering the energy gaps and ionic structures, we encourage the synthesis and laboratory detection of C_5_H_5_P^2+^, C_5_H_6_B^+^, and C_5_H_6_Al^+^, combining an ion trap experiment with spectroscopic techniques.^4^ The three GM candidates exhibit fingerprint bands related to the pentagonal-pyramidal geometry formation above 3000 cm^–1^. Experimental realization can be very challenging for borabenzene and aluminabenzene because their neutral counterparts have not been synthesized yet. Finally, the phosphorine dication GM candidate is the best choice for a laboratory detection, even forming a crystal structure similar to the benzene dication core.^12^

## Supplementary Material



## Notes

Details about the genetic algorithm, simulated infrared spectra, molecular parameters of GM candidates, and molecular geometries in XYZ format and frequencies and IR intensities (PDF)
