# Supplementary material for: New Molecular Geometries with Pentagonal-Pyramidal Structure
Source: ACS Omega. 2025 Dec 3;10(49):60450–6. doi: 10.1021/acsomega.5c07780 (PMC12713440; doi:10.1021/acsomega.5c07780)
Supplement: Supplementary file 1 [file ao5c07780_si_001.pdf]

Supporting Information: New Molecular Geometries With Pentagonal-Pyramidal Structure

Ricardo R. Oliveira,[*,†] Wania Wolff,[*,‡] and Amir L. Perlin[†]

†*Chemistry Institute, Federal University of Rio de Janeiro, Rio de Janeiro, RJ 21941-909, Brazil.*

‡*Physics Institute, Federal University of Rio de Janeiro, Rio de Janeiro, RJ 21941-909, Brazil.*

E-mail: rrodrigues.iq@gmail.com; wania@if.ufrj.br

Contents

S1 Details about the genetic algorithm **S-3**

S2 Simulated infrared spectra **S-4**

S3 Molecular parameters for the GM candidates **S-7**

S4 Molecular geometries in XYZ format **S-10**

S5 Frequencies (cm^{-1}) and IR Intensities (km mol^{-1}) **S-25**

References **S-61**

S1 Details about the genetic algorithm

The initial populations is constructed positioning the atoms in a cubic grid based on covalent atomic radii obtained from crystallographic data. The smallest radius is 0.3 Å (hydrogen atom). Combining the covalent radii, the next position is obtained and so one. This procedure reduces the chance of generating non-bonded structures. More details is present in the original AUTOMATON paper, methodology section and scheme 1.[S1] The initial population of 5N individuals was proposed, where N stand for the number of atoms. However, in our previous benchmark work based on benzene dication and derivatives, we found that 7N is necessary, instead.[S2] All generated structures were then optimized using the Gaussian software at DFT level. Also, the level of theory was PBE0/6-31G(d,p) based on our previous studies.[S2,S3]

The structure selection for the mating process is based on the exponential fitness function:

$$f = exp(-3\rho_i) \tag{S1}$$

where ρ_i is defined as:

$$\rho_i = \frac{E_i - E_{min}}{E_{max} - E_{min}} \tag{S2}$$

where E_i is the energy of i individual, and E_{min} and E_{max} are the minimum and maximum energy of all individuals. All individuals with $f \geq 0.5$ are selected for the mating process forming "α" isomers. The mating process is based on the "cut and splice" crossover operator (see scheme 2 in the original work)[S1]. Also, randomly individuals from "α" isomers are selected to be mutated. The mutation processes are: random atomic displacement and permutation of atomic positions (see scheme 3 in the original work)[S1]. After all operations, geometry optimizations were performed again. The 7N best individuals are selected to the new global optimization cycle. After nine cycles with the same global minimum candidate the global optimization is considered converged.

S2 Simulated infrared spectra

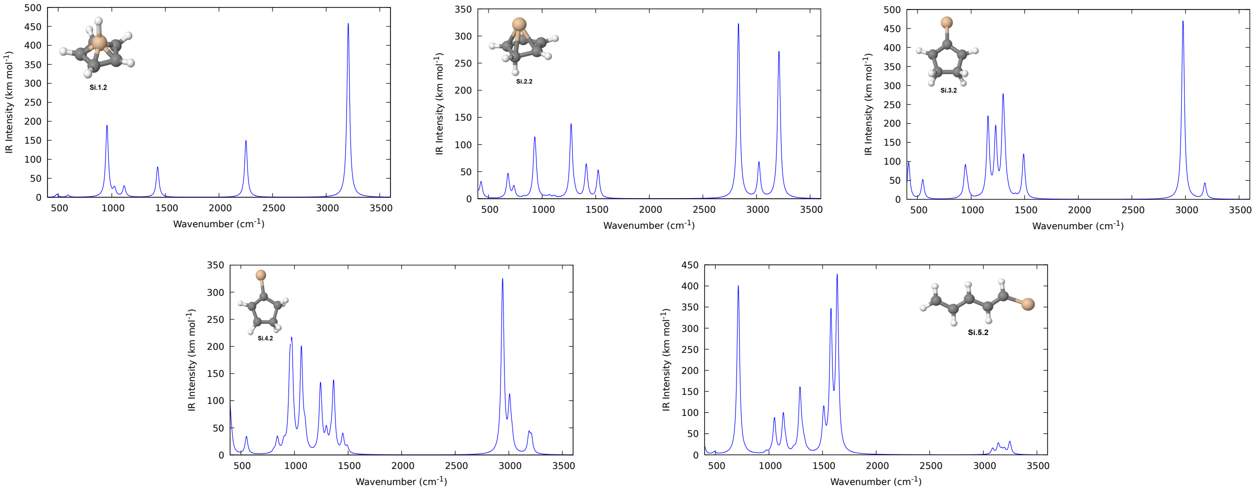

Figure S1: Simulated infrared spectra of $C_5H_6Si^{2+}$.

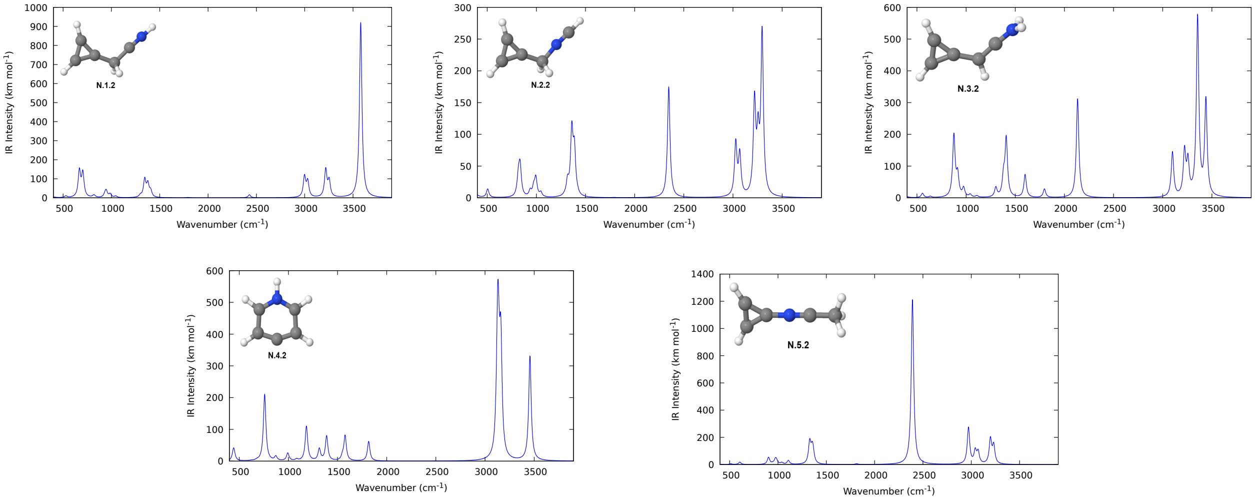

Figure S2: Simulated infrared spectra of $C_5H_5N^{2+}$.

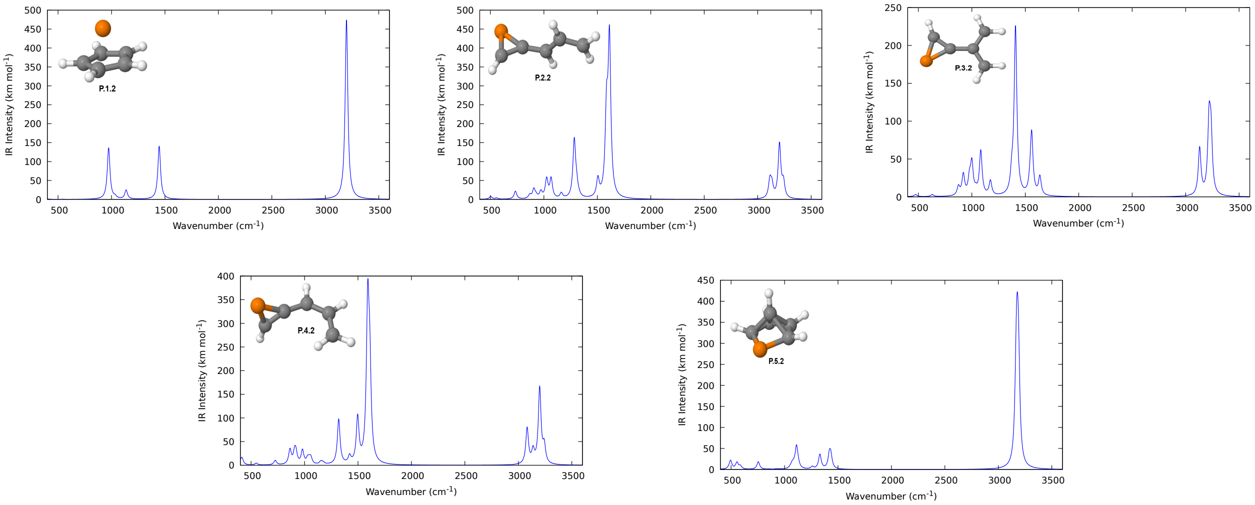

Figure S3: Simulated infrared spectra of $C_5H_5P^{2+}$.

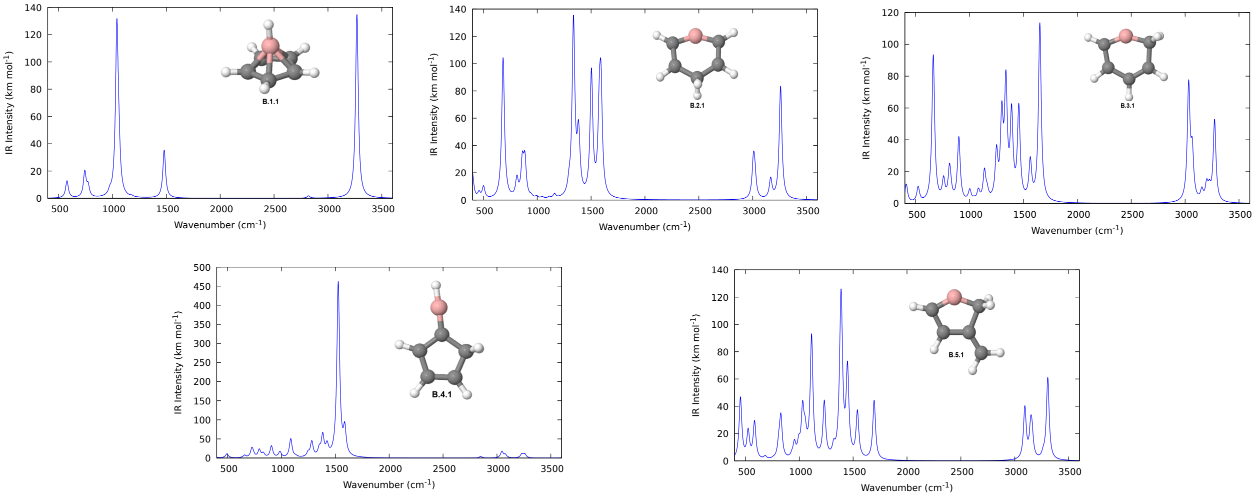

Figure S4: Simulated infrared spectra of $C_5H_6B^+$.

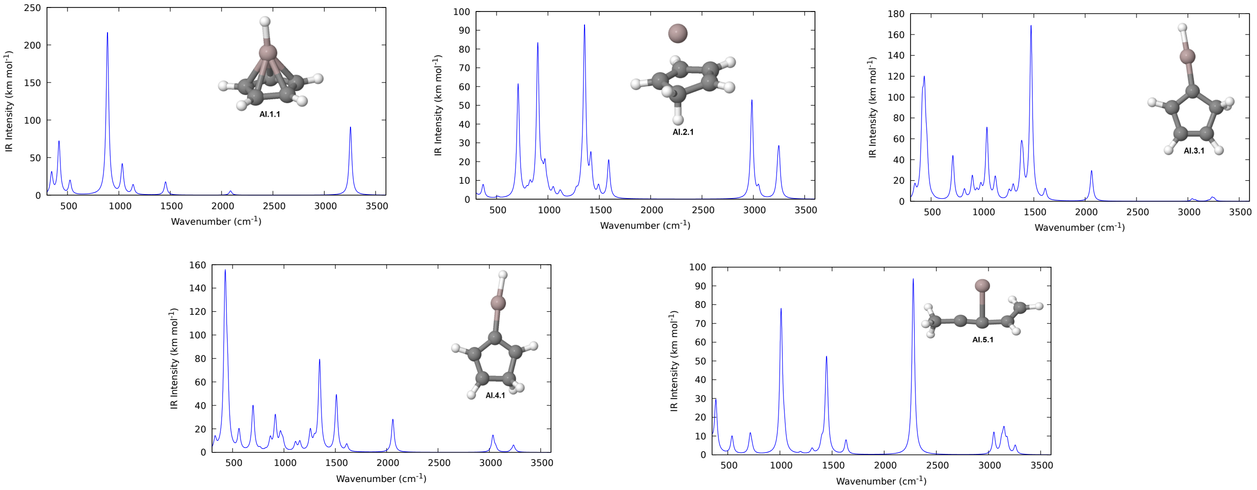

Figure S5: Simulated infrared spectra of $C_5H_6Al^+$.

S3 Molecular parameters for the GM candidates

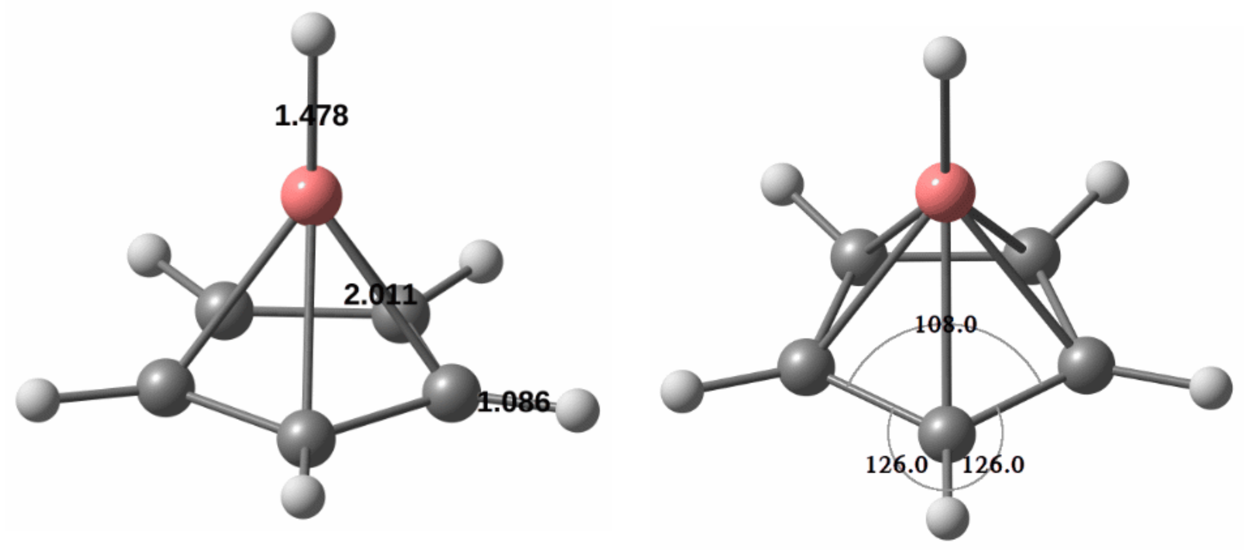

Figure S6: Geometric parameters of **Si.1.2**.

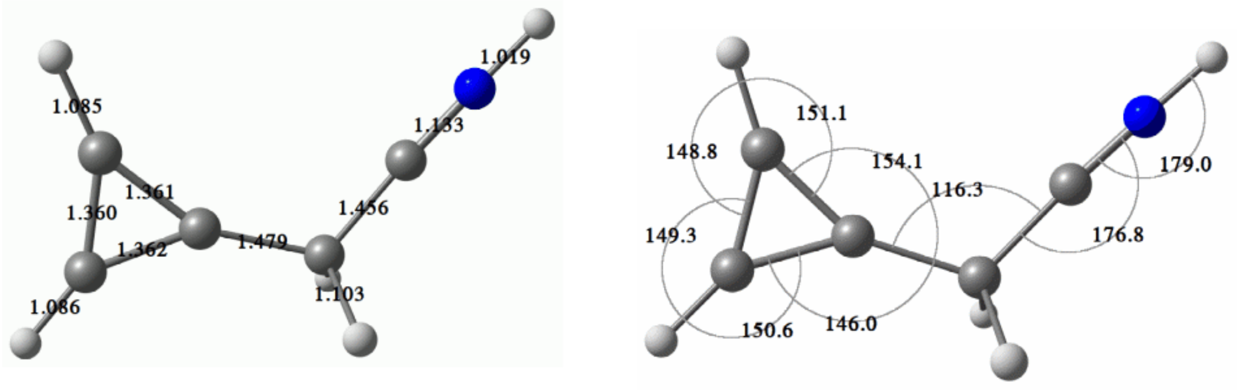

Figure S7: Geometric parameters of **N.1.2**.

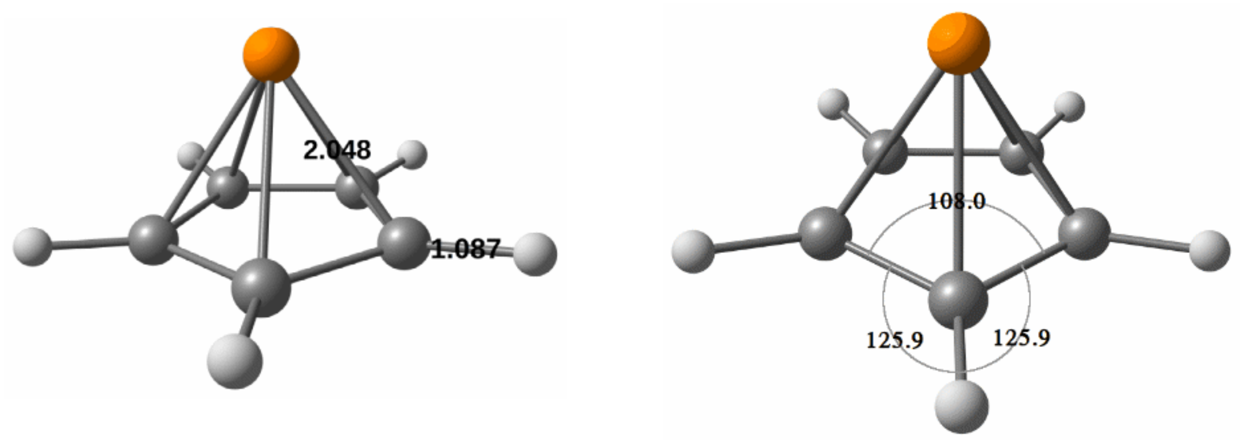

Figure S8: Geometric parameters of **P.1.2**.

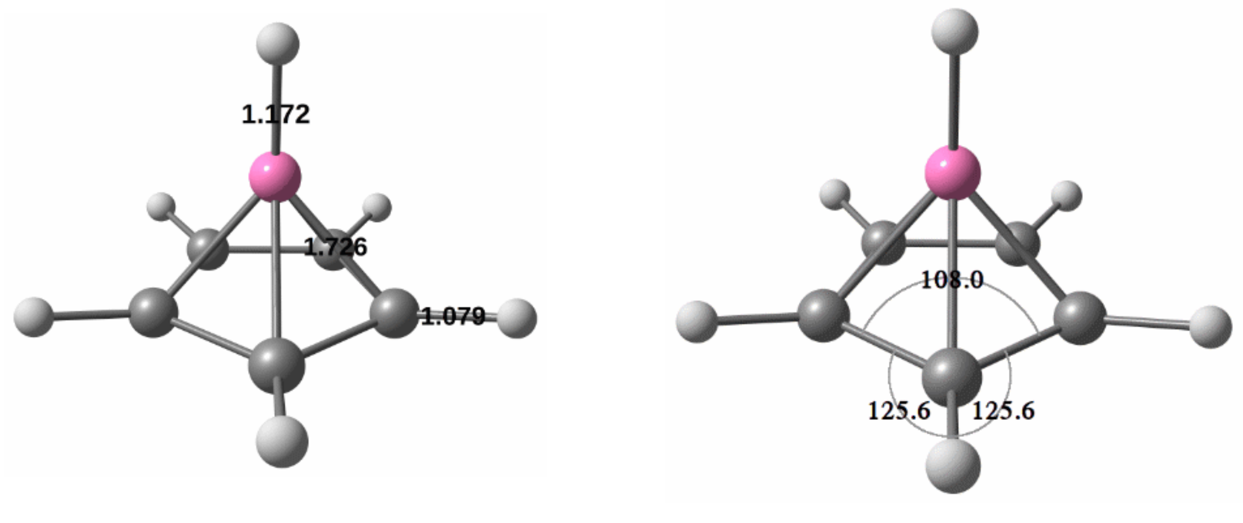

Figure S9: Geometric parameters of **B.1.2**.

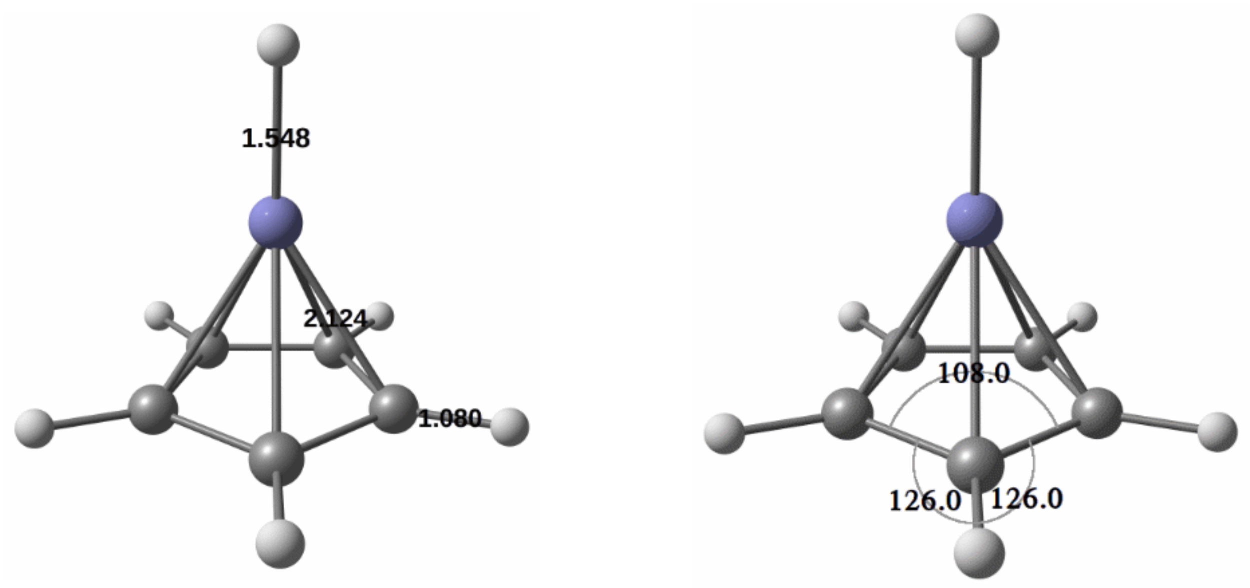

Figure S10: Geometric parameters of **Al.1.2**.

S4 Molecular geometries in XYZ format

```
12
Si_1_2
C    0.720922  -0.992264  -0.511609
C   -0.720922  -0.992264  -0.511609
C   -1.166476   0.379011  -0.511609
C    0.000000   1.226505  -0.511609
C    1.166476   0.379011  -0.511609
H    0.000000   2.312083  -0.473135
H    2.198921   0.714473  -0.473135
H    1.359008  -1.870514  -0.473135
H   -1.359008  -1.870514  -0.473135
H   -2.198921   0.714473  -0.473135
Si   0.000000   0.000000   1.082351
H    0.000000   0.000000   2.561018
12
Si_2_2
C         -0.146411 1.187823 -0.487188
C         0.400666 0.000019 -1.238223
C         -0.146392 -1.187812 -0.487216
C         -1.126632 -0.720997 0.380118
C         -1.126643 0.720972 0.380136
H         -1.760996 -1.340481 1.007345
H         -1.761016 1.340432 1.007378
H         0.085819 2.223227 -0.717408
H         1.431504 0.000032 -1.627406
```

H 0.085851 −2.223207 −0.717461

Si 1.071722 −0.000004 0.852901

H −0.212800 0.000023 −2.178820

12

Si_3_2

C 1.789878 −0.756112 0.000000

C 1.789879 0.756111 0.000000

C 0.388950 1.128667 0.000000

C 0.388948 −1.128667 0.000000

C −0.458414 0.000001 0.000000

H 2.305967 −1.228317 −0.856646

H 0.061402 −2.166973 0.000000

H 2.305967 1.228314 −0.856649

H 0.061405 2.166973 0.000001

Si −2.338723 0.000000 0.000000

H 2.305969 1.228315 0.856646

H 2.305966 −1.228316 0.856649

12

Si_4_2

C −1.688909 0.873993 0.000000

C −1.905730 −0.544841 −0.000001

C −0.528970 −1.180955 0.000000

C −0.302557 1.175709 0.000000

C 0.398681 −0.016059 0.000001

H −2.480978 1.620680 0.000001

H 0.095175 2.184890 −0.000001

H −2.558693 −0.833186 −0.849555

H −0.360372 −1.836794 −0.867823

Si 2.313489 −0.022336 0.000000

H −2.558694 −0.833185 0.849554

H −0.360373 −1.836792 0.867825

12

Si_5_2

C −3.516864 0.147088 0.000000

C −2.285686 −0.483341 0.000003

C −1.138654 0.286869 0.000000

C 0.192052 −0.276974 −0.000003

C 1.298604 0.512287 0.000000

H −4.442883 −0.423008 −0.000004

H −3.599130 1.231549 −0.000001

H −2.230459 −1.567307 0.000004

H −1.228038 1.371832 −0.000001

H 0.261278 −1.362925 −0.000006

Si 3.055462 −0.140363 0.000001

H 1.166053 1.599368 0.000003

11

N_1_2

C 1.584874 −0.108966 0.000000

C −2.267755 −0.218882 −0.000008

H 0.391131 −1.566849 0.877715

C −1.655539 0.995038 0.000005

C 0.362488 −0.899641 0.000006

H 3.462324 0.942081 −0.000009

H −3.182778 −0.803522 −0.000018

C −0.908072 −0.142662 0.000001

H −1.738710 2.077174 0.000012

N 2.568704 0.452666 −0.000005

H 0.391132 −1.566863 −0.877693

11

N_2_2

N 1.572908 −0.070086 0.000000

C −2.228512 −0.227829 −0.000001

H 0.446115 −1.542597 0.883867

C −1.625963 0.988757 −0.000001

C 0.402932 −0.890577 0.000003

H 3.503971 1.029397 −0.000003

H −3.136970 −0.822354 −0.000002

C −0.868496 −0.143070 0.000000

H −1.712798 2.070311 −0.000001

C 2.560575 0.489126 −0.000001

H 0.446116 −1.542604 −0.883856

11

N_3_2

C −1.467087 −0.173103 0.000000

C 2.317680 −0.299079 0.000001

C 1.777798 0.948953 0.000000

C −0.327584 −0.793815 −0.000001

H −3.088129 0.566991 0.874756

H 3.196853 −0.935480 0.000003

C 0.962864 −0.145015 0.000000

H 1.925110 2.023761 −0.000002

N −2.593212 0.348230 0.000001

H −0.375243 −1.887525 −0.000001

H −3.088130 0.566991 −0.874754

11

N_4_2

H −2.204376 −1.393188 −0.000001

C −1.267666 −0.832165 0.000000

C 0.000002 −1.196436 0.000001

C 1.267669 −0.832162 0.000000

H 2.204381 −1.393181 −0.000001

C 1.189132 0.580866 0.000000

H 2.102287 1.176162 −0.000001

N −0.000002 1.200476 0.000000

H −0.000002 2.224926 0.000000

C −1.189134 0.580863 0.000000

H −2.102291 1.176155 −0.000001

11

N_5_2

C −1.468730 0.002081 −0.002357

C 2.195400 −0.685159 0.000168

C 2.195715 0.684616 0.000218

N −0.312450 0.000401 −0.000189

H 2.747296 −1.622603 0.000201

C 1.021442 −0.000019 0.000064

H 2.748003 1.621831 0.000319

C −2.876349 0.001663 −0.001970

H −3.225686 −0.258291 1.012506

H −3.238532 −0.761401 −0.709215

H −3.248791 0.998564 −0.279221

11

P_1_2

C −0.501419 0.987267 0.717189

C −0.501419 0.987267 −0.717189

C −0.501419 −0.377036 −1.160447

C −0.501461 −1.220281 0.000000

C −0.501419 −0.377036 1.160447

H −0.422386 −2.304808 0.000000

H −0.422439 −0.712177 2.191904

H −0.422281 1.864613 1.354732

H −0.422281 1.864613 −1.354732

H −0.422439 −0.712177 −2.191904

P 1.143644 −0.000077 0.000000

11

P_2_2

C 1.872767 −0.076383 −0.453057

P −2.087501 −0.582146 −0.022318

H 1.090257 0.194399 1.527328

C −1.557588 1.041720 −0.142734

C 0.859572 0.115090 0.460796

H 3.398043 −0.053120 1.083626

H 1.671345 −0.164487 −1.515318

C −0.527931 0.208149 0.148027

C 3.172173 −0.134709 0.022186

H 4.011773 −0.261617 −0.658661

H −1.772867 2.093811 −0.313514

11

P_3_2

H −1.413552 2.188062 0.000091

C −1.218157 1.120175 0.000035

C 2.068373 1.022921 0.000066

C 1.730155 −1.323691 0.000004

H 1.733872 2.058990 0.000085

P −1.891567 −0.467776 −0.000069

H 3.144062 0.853364 0.000078

C 1.184315 −0.048065 0.000024

H 1.115538 −2.222361 −0.000029

H 2.809534 −1.465440 0.000022

C −0.267344 0.162665 0.000003

11

P_4_2

C −2.126288 0.369683 −0.169495

P 1.925506 −0.291439 −0.511141

H −0.770629 1.997840 −0.315338

C 1.355802 0.056109 1.065838

C −0.859096 0.921880 −0.119366

H −1.614064 −1.688627 0.307268

H −2.945552 1.043697 −0.398566

C 0.407811 0.323247 0.131983

H 1.524621 0.107509 2.139120

C −2.389191 −0.963087 0.073391

H −3.411197 −1.335823 0.040528

11

P_5_2

C	−0.063190	1.208850	−0.143725
C	1.262169	0.712324	−0.320241
C	1.262169	−0.712324	−0.320241
C	−0.063190	−1.208850	−0.143725
P	−1.387555	0.000000	−0.122677
H	−0.239768	−2.235562	0.175020
H	−0.239768	2.235562	0.175020
H	2.155609	1.312649	−0.153632
H	2.155610	−1.312649	−0.153632
C	0.338437	0.000000	0.900032
H	0.563283	0.000000	1.964784

12

B_1_1

C	0.710125	−0.977403	−0.227958
C	−0.710125	−0.977403	−0.227958
C	−1.149006	0.373335	−0.227958
C	0.000000	1.208137	−0.227958
C	1.149006	0.373335	−0.227958
H	0.000000	2.276522	−0.073127
H	2.165101	0.703484	−0.073127
H	1.338106	−1.841745	−0.073127
H	−1.338106	−1.841745	−0.073127
H	−2.165101	0.703484	−0.073127
B	0.000000	0.000000	1.005396
H	0.000000	0.000000	2.177399

12

B_1_1

C	0.710125	−0.977403	−0.227958
C	−0.710125	−0.977403	−0.227958
C	−1.149006	0.373335	−0.227958
C	0.000000	1.208137	−0.227958
C	1.149006	0.373335	−0.227958
H	0.000000	2.276522	−0.073127
H	2.165101	0.703484	−0.073127
H	1.338106	−1.841745	−0.073127
H	−1.338106	−1.841745	−0.073127
H	−2.165101	0.703484	−0.073127
5	0.000000	0.000000	1.005396
H	0.000000	0.000000	2.177399

12

B_2_1

H	2.185480	1.105644	−0.000002
C	1.284589	0.495766	0.000000
C	−0.000001	1.240207	0.000000
H	−0.000001	1.935339	−0.855179
C	−1.284590	0.495765	0.000000
H	−2.185481	1.105642	−0.000001
C	−1.390387	−0.863122	0.000000
H	−2.333158	−1.388670	−0.000002
5	0.000001	−1.267519	0.000001
C	1.390389	−0.863121	0.000000
H	2.333160	−1.388667	−0.000001

H −0.000001 1.935339 0.855180

12

B_3_1

H 2.128883 1.231520 −0.000001

C 1.204356 0.664522 −0.000001

C 0.006472 1.277254 0.000002

H −0.042946 2.358487 0.000003

C −1.252148 0.565858 0.000000

H −2.149743 1.182519 −0.000008

C −1.443020 −0.812872 0.000000

H −2.423265 −1.261465 −0.000006

5 −0.091327 −1.255961 0.000004

C 1.339005 −0.841120 −0.000002

H 1.907859 −1.176559 −0.878102

H 1.907856 −1.176553 0.878103

12

B_4_1

C −0.104463 −1.129100 0.000000

C 1.278397 −0.821075 0.000000

C 1.415967 0.531747 0.000000

C 0.100954 1.203076 0.000000

C −0.863213 0.045058 0.000000

H 2.073723 −1.552036 0.000000

H 2.352567 1.074885 0.000000

H −0.018776 1.853624 −0.875115

H −0.506523 −2.135152 0.000000

B −2.279617 0.000456 0.000000

H −3.449989 −0.075462 0.000000

H −0.018776 1.853625 0.875114

12

B_4_1

C −0.104463 −1.129100 0.000000

C 1.278397 −0.821075 0.000000

C 1.415967 0.531747 0.000000

C 0.100954 1.203076 0.000000

C −0.863213 0.045058 0.000000

H 2.073723 −1.552036 0.000000

H 2.352567 1.074885 0.000000

H −0.018776 1.853624 −0.875115

H −0.506523 −2.135152 0.000000

5 −2.279617 0.000456 0.000000

H −3.449989 −0.075462 0.000000

H −0.018776 1.853625 0.875114

12

B_5_1

C −1.768867 −0.425000 −0.000001

C −0.501958 −0.986033 0.000001

C 0.650372 −0.087506 0.000001

C 0.220309 1.394559 0.000000

5 −1.187185 0.884865 −0.000001

H −0.343821 −2.063548 0.000002

C 1.907566 −0.532630 −0.000001

H 0.541480 1.930289 −0.894912

H −2.712548 −0.941523 −0.000001

H 0.541476 1.930289 0.894913

H 2.107593 −1.598769 −0.000002

H 2.757215 0.138599 −0.000001

12

Al_1_1

C 0.000000 −1.212651 −0.530068

C −1.153300 −0.374730 −0.530068

C −0.712779 0.981055 −0.530068

C 0.712779 0.981055 −0.530068

C 1.153300 −0.374730 −0.530068

H 1.347837 1.855138 −0.530431

H 2.180846 −0.708600 −0.530431

H 0.000000 −2.293077 −0.530431

H −2.180846 −0.708600 −0.530431

H −1.347837 1.855138 −0.530431

Al 0.000000 0.000000 1.214658

H 0.000000 0.000000 2.763639

12

Al_2_1

C 0.499582 −0.338356 1.174866

C 0.463624 −1.262359 −0.000015

C 0.499582 −0.338327 −1.174874

C 0.768215 0.920213 −0.723485

C 0.768215 0.920195 0.723507

H 0.941993 1.790971 −1.341110

H 0.941993 1.790939 1.341153

H 0.470979 −0.660335 2.206297

H −0.292701 −2.057787 −0.000025

H 0.470980 −0.660282 −2.206312

Al −1.688456 0.169425 0.000002

H 1.421369 −1.814220 −0.000021

12

Al_3_1

C 1.049314 −0.524473 0.000000

C 0.562762 −1.877348 0.000000

C −0.785569 −1.845818 0.000000

C −1.272050 −0.444385 0.000000

C 0.000000 0.361919 0.000000

H 1.190022 −2.756830 0.000000

H −1.447129 −2.701345 0.000000

H −1.902899 −0.242723 0.874667

H 2.104822 −0.275061 0.000000

Al 0.307889 2.191040 0.000000

H 0.628783 3.715787 0.000000

H −1.902899 −0.242723 −0.874667

12

Al_3_1

C 1.049314 −0.524473 0.000000

C 0.562762 −1.877348 0.000000

C −0.785569 −1.845818 0.000000

C −1.272050 −0.444385 0.000000

C 0.000000 0.361919 0.000000

H 1.190022 −2.756830 0.000000

H −1.447129 −2.701345 0.000000

H −1.902899 −0.242723 0.874667

H 2.104822 −0.275061 0.000000

Al 0.307889 2.191040 0.000000

H 0.628783 3.715787 0.000000

H −1.902899 −0.242723 −0.874667

12

Al_4_1

C −0.312542 −1.072454 0.000002

C −1.771015 −0.798917 −0.000002

C −1.849277 0.686018 −0.000002

C −0.617504 1.214259 0.000001

C 0.358602 0.117432 0.000004

H −2.261272 −1.254336 −0.871124

H −2.778296 1.237846 −0.000004

H 0.102672 −2.073282 0.000004

Al 2.226712 0.028752 −0.000001

H 3.772198 −0.173701 −0.000008

H −0.370862 2.266006 0.000002

H −2.261276 −1.254337 0.871119

12

Al_5_1

C 2.659149 −0.288624 0.176905

C 1.239120 −0.482048 −0.025582

H 3.074186 −1.185535 0.644650

H 2.862022 0.559260 0.834183

H 3.167892 −0.129759 −0.775398

C 0.066439 −0.781169 −0.165822

C −1.321660 −0.939724 −0.389948

C −2.238269 −0.494886 0.489613

H −1.625706 −1.351038 −1.347909

H −1.960622 −0.142630 1.478692

H −3.295712 −0.543354 0.261881

Al −0.357749 1.593212 −0.123623

12

Al_5_1

C 2.659149 −0.288624 0.176905

C 1.239120 −0.482048 −0.025582

H 3.074186 −1.185535 0.644650

H 2.862022 0.559260 0.834183

H 3.167892 −0.129759 −0.775398

C 0.066439 −0.781169 −0.165822

C −1.321660 −0.939724 −0.389948

C −2.238269 −0.494886 0.489613

H −1.625706 −1.351038 −1.347909

H −1.960622 −0.142630 1.478692

H −3.295712 −0.543354 0.261881

Al −0.357749 1.593212 −0.123623

S5 Frequencies (cm^{-1}) and IR Intensities (km mol^{-1})

Si_1_2

Freq Int

387.4050 0.2514

387.4103 0.2510

487.3350 3.8108

487.3382 3.8132

582.1272 0.0000

582.1272 0.0000

591.9918 5.4405

855.2624 0.0000

855.2625 0.0000

942.5871 0.5255

942.5888 0.5254

955.5043 187.6132

993.4700 0.0000

993.4701 0.0000

1025.4120 9.9897

1025.4130 9.9921

1086.6905 0.0000

1086.6912 0.0000

1115.8187 28.3751

1268.1097 0.0000

1345.3639 0.0000

1345.3653 0.0000

1427.7229 40.0003

1427.7241 39.9948

2251.8299 149.4004

3202.0174 0.0000

3202.0177 0.0000

3206.2350 228.7201

3206.2353 228.7243

3217.5290 1.5621

Si_2_2

Freq Int

219.3698 0.2685

257.9848 5.0314

373.7773 41.2177

430.4630 28.7943

489.3997 0.0892

681.5703 44.7494

736.5683 20.5507

827.5078 1.8430

827.6985 0.3137

927.2549 45.9931

933.0226 58.9081

942.7477 19.3896

987.1600 0.7696

1030.7885 1.6619

1046.0811 0.7994

1068.9031 4.3351

1109.7674 2.8294

1123.1590 0.9945

1269.4374 3.9588

1271.0628 132.1326

1302.5863 5.2680

1410.2368 35.2788

1413.9125 27.3385

1523.4886 51.3987

2831.9133 322.1250

3023.3983 64.3996

3201.2688 1.8702

3205.7452 84.2913

3211.1595 180.5244

3218.5283 15.7051

Si_3_2

Freq Int

152.1142 0.7619

162.3195 4.2745

202.2904 0.0000

417.3106 96.6782

428.8758 0.4522

548.7837 50.7795

742.0623 0.0000

803.9732 0.0421

892.7031 2.2112

945.4646 79.2600

962.4693 2.2633

964.0855 22.5770

999.9414 0.0000

1061.1540 1.2823

1132.3288 0.7057

1157.9052 207.9829

1165.3403 0.0000

1229.1067 173.2223

1272.1473 1.2962

1298.3575 240.5016

1315.3568 58.4834

1343.7995 7.8639

1414.2555 4.8426

1490.2020 116.5669

2971.6654 60.7688

2977.5544 311.1280

2978.5416 0.0001

2979.6661 108.1590

3180.7554 39.5286

3181.5792 1.6510

Si_4_2

Freq Int

124.1573 3.7533

139.9081 1.2192

226.8275 0.0177

396.3509 94.3137

415.7376 13.2404

551.9908 32.5566

807.6815 4.1236

830.7764 1.1575

840.9554 27.1336

901.8302 15.8561

957.6160 126.6273

976.7055 155.2369

1001.7115 7.8348

1064.6121 186.4553

1098.8178 34.9077

1133.8277 0.3535

1138.0563 0.1826

1242.6992 116.8066

1253.3368 13.9723

1297.7459 34.9292

1337.7436 14.6679

1365.1912 129.2629

1451.0758 33.2162

1492.1182 11.3630

2937.1207 69.1574

2943.3589 259.3707

3007.4023 88.4677

3028.9939 22.6473

3187.7502 33.8706

3211.3225 28.3932

Si_5_2

Freq	Int
78.9151	5.7339
100.8339	1.9625
177.6887	0.5895
213.3703	0.7853
220.2180	2.3444
394.9488	22.7460
489.9638	3.9133
492.8534	1.9304
714.2545	399.5786
785.3886	1.2152
974.6387	4.0035
974.8329	0.8580
1016.7884	2.3667
1051.2793	82.5402
1134.0285	90.0944
1155.7815	13.4205
1230.8522	7.3511
1289.6794	150.3352
1315.9873	22.1937
1333.4827	10.6326
1363.2785	2.4972
1510.6762	94.3941
1578.2480	316.2118
1636.9861	407.5884
3086.6677	13.7410
3139.8018	24.1551

3159.0490 1.2200

3171.6072 7.9964

3196.8204 11.2375

3247.2223 30.4417

N_1_2

Freq Int

77.1057 0.0728

137.9954 2.1987

344.9384 27.3536

366.9945 4.5040

413.1160 1.4338

528.0353 8.3081

668.2287 137.7715

703.9687 125.5621

807.1170 1.9181

820.8545 11.4034

933.6984 16.4674

946.5452 30.9704

986.4166 18.2486

1022.2707 0.0678

1044.8790 7.6037

1243.1531 0.0109

1299.3093 10.6504

1344.4497 93.8582

1376.9198 67.3286

1406.3135 31.1015

1792.8044 1.8863

2427.2612 15.7453

2997.5482 107.8718

3030.9271 81.3134

3217.2498 144.2445

3252.8486 83.5314

3579.4481 918.8425

N_2_2

Freq Int

99.3751 1.2112

146.5907 7.9313

328.9178 48.0475

332.3046 17.6925

386.8579 3.0179

504.8095 13.3448

806.0520 2.0187

817.5299 27.9694

832.7894 24.9534

836.4583 22.2865

937.4079 9.4338

973.1537 13.6884

994.6370 29.2189

1019.8596 0.0811

1045.6407 6.9215

1285.7712 1.2413

1317.0485 22.4954

1360.4789 101.1397

1386.8630 69.1788

1418.1644 2.4340

1792.8971 0.3569

2346.7516 174.4587

3028.1755 83.4310

3069.1402 64.4186

3219.2754 145.4100

3255.5155 83.9125

3296.8587 254.9237

N_3_2

Freq Int

78.0623 3.5867

135.6157 5.1591

348.7325 27.2144

379.7380 2.1963

395.3452 1.0499

556.0876 13.5441

635.1218 3.8544

838.6913 0.3840

875.6868 193.0716

886.9742 1.3207

913.9673 66.1134

974.5739 27.2967

1015.6480 0.0094

1041.4257 8.2865

1109.6605 4.5809

1119.9119 0.1909

1302.3218 29.4853

1380.8827 58.6083

1408.4477 182.0100

1600.2957 72.0887

1796.6345 26.8526

2134.1266 311.1288

3099.2727 140.4984

3221.7616 138.5500

3256.6808 102.7613

3354.7900 564.1247

3439.1636 300.3117

N_4_2

Freq Int

418.1457 0.0000

440.8830 27.6719

447.6245 15.4382

564.5243 0.0000

622.7306 0.8446

680.5662 1.8537

758.4239 209.7238

871.7848 13.2234

918.7475 0.0881

961.3871 0.0000

991.2112 4.2655

994.1522 19.9376

1017.9899 0.3370

1083.3848 4.7240

1182.1592 0.0141

1183.1987 109.1488

1281.5374 0.0011

1314.4439 37.0657

1388.8284 77.9675

1549.7084 11.8572

1576.7582 78.6183

1816.4531 61.7747

3127.3140 313.1222

3136.4921 230.1355

3160.1991 325.0717

3166.3094 20.4307

3457.0143 328.6324

N_5_2

Freq Int

15.9842 0.3021

124.5977 10.2383

128.2199 13.3322

380.1615 0.0113

407.0411 2.0147

510.2441 2.3224

510.3710 3.2991

605.0688 17.5015

902.0001 51.0426

944.7888 5.4318

972.6613 28.9480

983.5728 24.9287

1006.9828 0.4129

1039.4223 12.0265

1107.2260 28.4401

1328.1561 157.9015

1352.2404 65.2476

1355.5370 7.0463

1359.5462 40.4113

1365.6581 38.9571

1813.9934 5.3960

2391.4307 1210.3606

2971.1696 268.4749

3040.8068 92.4609

3070.5754 84.6702

3198.0553 180.4723

3231.9823 131.5452

P_1_2

Freq Int

376.1495 2.2773

376.2528 2.2787

534.3780 0.0001

537.6930 0.0002

547.6713 0.2864

838.9736 0.0000

839.8597 0.0000

973.0694 133.2254

976.0496 0.1688

976.1303 2.3515

1018.1110 0.0003

1018.7478 0.0003

1033.4853 2.5576

1033.9357 2.6047

1105.8801 0.0001

1105.9269 0.0000

1136.8074 23.6275

1281.0759 0.0001

1380.8868 0.0064

1381.2186 0.0029

1445.8735 70.2569

1446.5059 70.1584

3193.1846 0.0005

3193.2720 0.0605

3198.1691 236.6013

3198.2494 236.5960

3210.2792 0.2991

P_3_2

Freq Int

106.1789 0.9190

127.5156 5.3790

164.9821 2.5691

284.8618 1.2224

382.6647 0.8254

502.4553 7.2676

556.0216 3.0750

733.4543 20.8384

868.1434 9.9261

906.2357 24.2581

927.6900 6.5577

971.8490 17.9810

1025.1106 46.7620

1032.6651 4.7675

1067.8315 51.3954

1162.9792 13.5590

1284.2096 152.3868

1306.2981 27.7608

1329.6014 3.5890

1505.3647 47.4518

1586.2082 209.1935

1613.3238 409.7588

3114.0722 46.2260

3132.9828 33.0171

3202.7683 14.6672

3204.0645 128.4505

3240.9133 40.8428

P_3_2

Freq	Int
49.0957	0.1667
180.9311	0.1535
272.7257	26.0118
315.9627	0.5049
365.4133	0.0413
474.1324	2.6483
629.3996	2.5823
633.1117	0.0250
650.1303	0.0282
874.9018	11.7693
920.3042	27.5485
978.0364	20.5528
1000.2649	40.9471
1038.0246	4.9361
1083.6347	58.7364
1170.5022	0.5154
1173.8907	18.8036
1374.2820	22.6467
1408.6824	221.0004
1544.1154	8.0844
1559.7025	81.2209
1636.3415	24.6497
3129.9719	31.6706
3132.0984	31.1604
3219.2684	91.0141
3235.1077	21.3466

3237.9965 53.4702

P_4_2

Freq Int

81.6648 0.9866

143.4848 0.9929

170.6968 0.4073

369.2741 3.5698

385.1804 7.5986

414.2817 14.6892

548.7129 3.9612

725.7503 9.3498

863.7927 31.0550

907.7353 24.0191

920.1624 19.4192

980.8535 29.4933

1028.5452 10.5015

1042.5563 6.9042

1058.4033 13.5641

1152.2921 6.0582

1173.6230 4.0843

1319.7802 95.4412

1421.6243 15.1238

1496.9662 96.1616

1591.1759 319.6853

1610.1323 176.9275

3082.4931 76.2582

3137.9051 26.8680

3198.4988 38.8430

3200.1907 122.4163

3242.1220 36.5365

P_5_2

Freq Int

366.1668 3.2643

475.2740 1.0471

494.8475 20.6740

554.4871 15.5192

586.6433 7.2706

720.4713 0.0954

754.4613 18.0361

837.9580 0.0834

858.8691 0.4905

925.9542 1.1038

955.0059 0.4482

1049.8720 1.8236

1062.2237 1.5972

1071.3348 9.4385

1088.3289 6.9159

1110.9330 0.0226

1111.7482 50.9888

1127.0491 8.0809

1259.4390 5.4475

1330.5677 34.7949

1419.2576 30.9687

1433.0412 27.9494

3166.0186 97.0072

3168.2283 49.3609

3174.7436 149.2069

3181.8083 86.0872

3189.4248 166.3852

B_1_1

Freq Int

578.6861 6.2627

578.6877 6.2633

597.8716 0.0000

597.8725 0.0000

743.6673 18.6091

774.6016 3.9926

774.6031 3.9942

873.2803 0.0000

873.2824 0.0000

976.1102 1.4148

976.1111 1.4152

1008.0380 0.0000

1008.0383 0.0000

1041.5319 122.5636

1060.5165 11.1988

1060.5170 11.2007

1106.3772 0.0000

1106.3774 0.0000

1178.8761 0.6278

1286.1157 0.0000

1406.6910 0.0000

1406.6942 0.0000

1480.8716 17.5671

1480.8729 17.5640

2819.4510 1.7027

3260.7054 0.0000

3260.7057 0.0000

3268.8306 67.1063

3268.8307 67.1076

3277.6407 0.7114

B_2_1

Freq Int

269.7293 5.8206

297.7753 0.0000

398.9795 19.1971

460.0418 4.2017

502.1163 8.8153

683.7676 103.3878

697.5161 0.0000

811.8489 13.5425

862.4599 25.5398

885.8303 26.7702

944.1474 1.5209

995.5090 1.4823

1007.3796 0.0000

1046.8903 1.1450

1111.5676 1.1083

1161.1749 2.9805

1191.0722 0.0000

1295.0676 6.5382

1336.8621 129.2791

1382.0783 34.1209

1389.5076 12.1914

1503.3326 90.8259

1576.7172 51.9874

1591.8448 71.9637

3006.8415 27.1811

3020.0022 13.6942

3166.0772 8.2711

3166.6613 6.0654

3258.2273 63.3567

3258.7982 19.4990

B_3_1

Freq Int

226.3259 5.5330

342.6331 1.2977

412.8523 1.3590

413.0061 10.0819

523.3561 9.2604

663.5364	92.4066
759.3206	12.9088
813.7741	19.4119
825.6432	4.0167
899.8053	25.2029
902.3919	15.2195
981.8296	0.3541
1002.2470	6.7356
1042.1689	1.1587
1082.0494	6.4821
1138.2854	18.3272
1163.1924	5.0901
1250.3209	28.7461
1299.2375	49.7891
1337.3223	71.1064
1390.0511	52.1981
1456.9940	57.3675
1563.6837	24.1520
1652.1389	112.0509
3033.0367	72.3686
3066.4543	28.5439
3155.6155	6.4202
3200.1914	10.0473
3225.4860	7.0691
3273.4629	51.3516

B_4_1

Freq	Int
173.3128	3.6312
175.0198	5.7902
354.1375	17.5651
494.6937	10.6065
658.3947	6.0115
725.9168	22.0458
738.1718	6.8545
795.0671	20.1259
831.8508	10.3569
907.3661	3.4493
907.6455	26.7449
972.7325	0.6768
986.4949	14.2980
1023.5364	0.0379
1086.8631	49.0708
1133.9353	2.9773
1145.6820	0.0001
1247.4941	10.5858
1281.9199	39.6667
1351.5332	18.9434
1383.3884	54.2939
1424.0385	27.3487
1527.4554	456.6644
1587.8383	67.7379
2848.8524	3.7160
3046.0879	16.4399

3077.6346 7.6973

3222.3990 0.6271

3231.8864 9.5302

3258.6175 9.5358

B_5_1

Freq Int

177.2729 0.9740

332.9731 0.1868

352.6133 1.0363

454.6229 45.4644

526.3059 20.0837

585.4124 27.3958

684.1089 2.2020

758.4510 0.5668

810.6823 9.3289

829.7195 30.2188

933.0241 2.0227

955.7100 10.4668

995.8243 9.6185

1031.4884 33.9905

1054.5915 14.9065

1072.0761 1.8074

1114.6958 89.2971

1232.8559 40.7537

1320.0533 7.2442

1381.6750 31.4912

1390.0328 95.5155

1447.8985 63.9138

1540.1506 33.8654

1695.2575 43.3701

3093.4249 37.9000

3147.9522 20.2075

3159.0569 11.5525

3162.2832 3.6815

3263.9136 3.2995

3306.2963 60.2088

Al_1_1

Freq Int

345.2363 14.1187

345.2365 14.1200

417.3476 35.2259

417.3496 35.2315

524.7382 18.1290

629.2226 0.0000

629.2244 0.0000

871.1458 0.0000

871.1471 0.0000

875.8186 2.7951

875.8250 2.7949

889.2296 213.0606

955.4935 0.0000

955.4948 0.0000

1033.1319 19.6806

1033.1322 19.6756

1085.1599 0.0000

1085.1601 0.0000

1138.7624 12.5929

1279.6275 0.0000

1377.0823 0.0000

1377.0830 0.0000

1454.7830 8.7387

1454.7831 8.7354

2087.2150 5.5855

3245.5376 0.0000

3245.5380 0.0000

3255.4119 45.2306

3255.4121 45.2253

3264.7736 0.1802

Al_2_1

Freq Int

148.5914 7.8480

171.8940 6.8670

228.9455 65.8285

369.2823 6.8242

510.8063 0.8102

709.9734 60.4723

794.2669 2.4495

823.1336 1.8374

828.0049 3.5687

901.1777 81.1678

945.6186 7.5304

971.4416 14.4740

995.8894 3.1353

1008.0826 0.1797

1050.7603 3.3111

1056.9077 1.2579

1118.1843 3.0260

1133.9199 0.9835

1275.9753 2.7503

1326.4310 4.4892

1356.5890 90.5632

1418.6145 19.6207

1493.0911 5.5526

1591.6142 20.2926

2985.9983 52.4874

3050.9960 5.0820

3230.2254 0.4104

3237.9897 9.9492

3250.1294 21.3803

3254.9766 0.4736

Al_3_1

Freq Int

131.9328 0.3717

141.3208 4.1045

341.5038	11.5880
414.6779	71.6479
435.4297	84.3550
458.7038	33.4141
517.2161	0.6061
711.8741	42.7158
824.5794	9.5528
858.0505	0.7366
901.5798	22.0922
946.1118	6.6513
983.7043	11.2512
1003.8878	0.7974
1043.5616	68.5926
1123.5997	16.8644
1132.0870	5.1689
1142.3000	0.0481
1259.9027	7.7387
1299.5335	12.0553
1377.8796	38.6456
1392.8534	26.4382
1473.5514	166.4643
1611.5429	10.1102
2062.6979	29.3434
3041.6125	2.2524
3071.7849	1.2076
3198.6717	0.6548
3235.1820	3.3387

3256.5276 2.2183

Al_4_1

Freq Int

110.1843 1.3818

129.3520 7.3535

329.5628 9.8549

423.3418 67.4519

431.0729 83.9833

451.3635 43.1782

562.4943 17.1324

699.1704 38.7655

767.2242 1.7937

820.0990 0.8037

867.2617 10.1726

915.9150 29.4622

965.3019 12.0213

977.3963 1.8320

989.6278 7.0180

1112.3022 7.0416

1118.8344 0.1971

1154.3870 7.8136

1256.9507 16.8013

1296.6893 7.1760

1347.5837 77.5878

1379.9054 0.2622

1510.4321 48.2354

1611.5645 5.9342

2060.3353 28.1164

3034.6774 13.8951

3061.4903 3.2990

3210.0642 0.9711

3235.1819 5.7048

3255.8491 0.4616

Al_5_1

Freq Int

55.8043 0.0749

95.8839 1.2840

103.8225 3.7421

136.8040 4.6537

229.2059 23.7831

300.2542 73.7852

311.5148 5.6641

385.7939 26.7155

540.8967 9.2853

715.0250 10.0725

731.5811 2.6780

1010.2995 51.7257

1013.2264 22.1535

1022.7617 4.7391

1041.3674 3.8827

1044.8827 3.7961

1197.8968 0.8550

1308.4852 2.6269

1400.9130 5.3320

1444.5452 21.3717

1446.7470 13.7700

1451.3944 18.5231

1633.8294 7.5162

2278.8031 93.7455

3051.6207 11.4816

3125.6013 5.6649

3138.5996 1.7809

3149.5886 10.6674

3179.1349 6.4735

3256.7476 4.6115

```
12
Si six−membered ring
Si      1.413645 0.000000 −0.000010
C       0.324432 −1.511825 0.000000
C       −1.014407 −1.305641 0.000013
C       −1.625704 0.000000 −0.000002
C       −1.014407 1.305641 0.000013
C       0.324432 1.511825 0.000000
H       2.889496 0.000000 −0.000011
H       0.729966 −2.522248 −0.000002
H       −1.694884 −2.154185 0.000021
H       −2.716761 0.000000 −0.000030
H       −1.694884 2.154186 0.000021
```

H 0.729966 2.522249 −0.000002

12

Si transition state

Si 1.517597 −0.141342 −0.197871

C 0.468763 1.228657 0.287021

C −0.878855 1.265197 −0.002077

C −1.772180 0.137805 −0.085782

C −1.223568 −1.105252 −0.090703

C 0.138406 −1.273475 0.245200

H 2.985866 −0.298380 −0.259327

H 1.014763 2.150569 0.530012

H −1.300078 2.237066 −0.282001

H −2.838508 0.278471 −0.227615

H −1.844072 −1.997972 −0.240835

H 0.340271 −1.908563 1.128003

11

N six−membered ring

H −1.174082 −2.122264 0.156548

H 1.310522 −1.990321 −0.200949

H −1.074692 1.971306 0.687022

C −0.672540 −1.153333 0.061445

N 0.612461 1.085317 −0.097367

C 0.724210 −1.112256 0.098473

C −0.644205 1.169473 0.057213

H −2.525501 0.001740 −0.191361

C 1.473261 0.169159 0.037934

C −1.438183 −0.022237 −0.172725

H 2.521270 0.237482 −0.263736

11

N transition state

N −0.688558 0.000008 0.899666

C −1.095889 0.000013 −0.445455

C −0.220135 1.162606 −0.162280

C 1.116824 0.725798 −0.083369

C 1.116804 −0.725824 −0.083369

C −0.220164 −1.162601 −0.162279

H −2.123800 0.000022 −0.829908

H −0.614765 2.164699 −0.005492

H 1.994343 1.353938 0.081878

H 1.994308 −1.353987 0.081866

H −0.614820 −2.164685 −0.005489

11

N pentagonal−pyramidal

C 0.234879 −0.984276 0.715017

C 0.234879 −0.984276 −0.715017

C 0.234879 0.375875 −1.157006

C 0.234999 1.216541 0.000000

C 0.234879 0.375875 1.157006

H −0.036698 2.272372 0.000000

H −0.036844 0.702084 2.161156

H −0.036827 −1.838469 1.335592

H −0.036827 −1.838469 −1.335592

H −0.036844 0.702084 −2.161156

N −0.980435 0.000281 0.000000

11

P six−membered ring

P −1.246261 −0.619223 −0.153548

C −1.070893 1.034374 0.340451

C 0.179073 1.420318 −0.091910

C 1.442089 0.725538 −0.005604

C 1.581780 −0.651083 −0.091523

C 0.389233 −1.308126 0.156503

H −1.936523 1.695756 0.306648

H 0.224798 2.399141 −0.595380

H 2.317051 1.345711 0.217939

H 2.548552 −1.141406 −0.012616

H 0.412350 −2.336987 0.539136

11

P transition state

P 1.016668 −0.646600 −0.392894

C 1.137377 0.599530 0.796756

C 0.274129 1.174396 −0.231696

C −1.163094 0.985867 −0.185653

C −1.646471 −0.300271 0.115764

C −0.667183 −1.219267 0.248555

H 1.740039 1.063966 1.586626

H 0.640114 2.108638 −0.671773

H −1.810329 1.843711 −0.377473

H −2.701007 −0.506861 0.296726

H −0.727379 −2.251986 0.596945

12

B six−membered ring

5 0.000000 −1.377068 0.150267

C 1.343384 −0.723660 −0.175046

C 1.265257 0.630996 0.013092

C 0.000000 1.264962 0.084010

C −1.265257 0.630996 0.013092

C −1.343384 −0.723660 −0.175046

H 0.000000 −2.036253 1.159173

H 2.302715 −1.207027 −0.323022

H 2.153128 1.253315 0.024101

H 0.000000 2.351217 0.126726

H −2.153128 1.253315 0.024101

H −2.302715 −1.207027 −0.323022

12

B transition state

5 −1.488569 −0.636609 −0.319996

C −0.236375 −1.236444 0.179072

C 1.122170 −0.875926 −0.089073

C 1.335056 0.451682 −0.097599

C 0.162631 1.307035 0.032581
C −1.085800 0.814949 0.175015
H −2.554332 −0.920837 −0.737744
H −0.387999 −1.765386 1.135656
H 1.915985 −1.615894 −0.082034
H 2.332410 0.867499 −0.159594
H 0.302127 2.382881 −0.033979
H −1.951437 1.467014 0.277703

12
Al six−membered ring
Al −1.563853 0.000000 0.000000
C −0.304989 −1.542614 −0.000001
C 1.034785 −1.297913 0.000000
C 1.615428 0.000000 0.000001
C 1.034785 1.297913 0.000000
C −0.304989 1.542614 −0.000001
H −3.125887 0.000000 0.000005
H −0.595553 −2.591288 −0.000002
H 1.746421 −2.118989 0.000000
H 2.704125 0.000000 0.000002
H 1.746421 2.118989 0.000000
H −0.595553 2.591288 −0.000002

12
Al transition state
Al −1.673039 −0.112617 −0.195800

C	−0.197447	−1.249415	0.276289
C	1.158471	−1.150864	−0.087862
C	1.754863	0.055889	−0.215329
C	0.959385	1.248027	0.001333
C	−0.323073	1.230191	0.390476
H	−3.164886	−0.018986	−0.627789
H	−0.345029	−1.770706	1.235476
H	1.763153	−2.057653	−0.061830
H	2.817763	0.130621	−0.403478
H	1.448348	2.203267	−0.184501
H	−0.883030	2.174503	0.398082

References

(S1) Yañez, O.; Báez-Grez, R.; Inostroza, D.; Rabanal-León, W. A.; Pino-Rios, R.; Garza, J.; Tiznado, W. AUTOMATON: A Program That Combines a Probabilistic Cellular Automata and a Genetic Algorithm for Global Minimum Search of Clusters and Molecules. *J. Chem. Theory Comput.* **2019**, *15*, 1463–1475.

(S2) Wolff, W.; Perlin, A.; Oliveira, R. R.; Fantuzzi, F.; Coutinho, L. H.; de A Ribeiro, F.; Hilgers, G. Production of Long-Lived Benzene Dications from Electron Impact in the 20–2000 eV Energy Range Combined with the Search for Global Minimum Structures. *J. Phys. Chem. A* **2020**,

(S3) Perlin, A. L.; Wolff, W.; Oliveira, R. R. Low Energy Isomers and Infrared Spectra Simulations of C4H3N, C4H4N, and C4H5N and Related Ions. *Journal of Physical Chemistry A* **2023**, *127*, 2481–2488.
